# COVID-19-Related Suicides in Bangladesh Due to Lockdown and Economic Factors: Case Study Evidence from Media Reports

**DOI:** 10.1007/s11469-020-00307-y

**Published:** 2020-05-15

**Authors:** A. K. M. Israfil Bhuiyan, Najmuj Sakib, Amir H. Pakpour, Mark D. Griffiths, Mohammed A. Mamun

**Affiliations:** 1 Undergraduate Research Organization, Savar, Dhaka, Bangladesh; 2 Department of Microbiology, Jashore University of Science and Technology, Jashore, Bangladesh; 3 Department of Nursing, School of Health and Welfare, Jönköping University, Jönköping, Sweden; 4 Psychology Department, Nottingham Trent University, 50 Shakespeare Street, Nottingham, NG1 4FQ UK; 5 Department of Public Health & Informatics, Jahangirnagar University, Savar, Dhaka, Bangladesh

The incidence and mortality of the coronavirus-2019 disease (COVID-19) have increased dramatically around the world. The effects of COVID-19 pandemic are not limited to health, but also have a major impact on the social and economic aspects. Meanwhile, developing and less developed countries are arguably experiencing more severe crises than developed countries, with many small and medium-sized businesses being disrupted and even bankrupt (Fernandes 2020). Consequently, some individuals’ mental health is very fragile (Lin 2020). Sahoo et al. (2020) reported some of the psychological consequences in India (the neighboring country of Bangladesh) including self-harm due to COVID-19 misinformation. Moreover, impacts on mental health (e.g., depression, anxiety, panic, and traumatic stress) can also occur due to the lack of accurate information (Rajkumar 2020; Sahoo et al. 2020; Tandon 2020).

In addition, pandemic-related restraints (e.g., spatial distancing, isolation, home quarantine, etc.) is impacting on economic sustainability and well-being, which may induce psychological mediators, such as sadness, worry, fear, anger, annoyance, frustration, guilt, helplessness, loneliness, and nervousness (Mukhtar 2020; Mamun and Griffiths 2020a). These mediators are also distinctive features of psychological suffering that individuals can experience during and after pandemics (Ahorsu et al. 2020; Pakpour and Griffiths 2020). Without early economic interventions, such mental health issues can facilitate suicidal behaviors among some individuals (Arafat and Mamun 2019; Mamun and Griffiths 2020b, c; Jahan et al. 2020), because economic recession, unemployment, and poverty are strongly associated with severe psychological comorbidities such as suicidal behaviors (Goldman-Mellor et al. 2010; Oyesanya et al. 2015; Rafi et al. 2019). There is one prior study that has examined COVID-19-related suicide in Bangladesh (Mamun and Griffiths 2020a).

## Cases

The first published case study of COVID-19-related suicide in Bangladesh raised the possibility of further suicides (Mamun and Griffiths 2020a). Although this suicide occurred due to COVID-19 fear and xenophobia, the causes of consequent COVID-19 suicides have not been investigated in the country. Therefore, we briefly present eight additional suicide-related cases that occurred in Bangladesh during 3 weeks in April 2020, since the country lockdown (as a result of the COVID-19 pandemic) took effect.

### Case 1

On 6 April, an adult man (aged 30 years) from Mohespur Upazila in Jhenaidah committed suicide (by hanging himself) due to the pressure of unpaid debts. In addition, his family was half-fed and had starved for a week after losing work after the lockdown and was denied any financial support from local government authorities (United News of Bangladesh 2020).

### Case 2

On 10 April, a female adolescent (aged 10 years) from Belkuchi municipality of Sirajgonj committed suicide (by hanging herself) because she was rebuked by her father for asking for food. The lockdown meant that the girl’s father had to close his small loom factory and the family therefore had no money. The whole family had starved for a couple of days and they were also denied any financial relief from the local government authorities (Kaler Kantho 2020a).

### Case 3

On 12 April, a woman and mother of five children (aged 35 years) from Cox’s Bazar attempted suicide by hanging, although one of her sons rescued her by getting help from her neighbors. Her husband lost his job because of the lockdown and they were also ineligible to receive relief goods from the local government authorities. The mother could not bear to see her starving children’s faces and thought that by killing herself she could provide more food for her starving children (Campus Today 2020).

### Case 4

On 13 April, a young adult man (aged 27 years) from Noldangga village in Natore committed suicide (by hanging himself). He was a day laborer and he became unexpectedly unemployed as a result of the lockdown. He was struggling with starvation and to compound the situation, his wife also left him (prior to the pandemic) and the loneliness made his living situation worse (Kaler Kantho 2020b).

### Case 5

On 14 April, a woman (whose age was not reported) from Dhamrai in Dhaka attempted suicide and kill her two children by setting themselves on fire with kerosene oil. Her husband became unemployed due to the shutdown of a garment factory where he worked and the mother was unable to work in a tea shop where she and her father-in-law worked. Consequently, the family experienced economic hardship. Additionally, she was asked by her father-in-law to leave the house with husband and children (RisingBD 2020a).

### Case 6

On 16 April, an adult man (aged 30 years) from Bashkhali Upazila in Chattogram committed suicide (although no details of how were reported). The man was an auto-rickshaw driver and was unable to earn any money for his family because he was unable to use his vehicle to earn money during the lockdown. He approached the local government authorities for financial relief but was denied because they claimed there were other more deserving cases for financial help than his own (Daily Star 2020a).

### Cases 7 and 8

On 24 April, a poverty-stricken husband (aged 30 years) and wife (aged 24 years) from Keshapur committed suicide both hanging themselves from the roof of their house due to lockdown-related economic distress. The couple had a 3-year-old child and the family were very poor. The local government authority reported that the suicides were due to existing debts made worse by the national lockdown (Manab Zamin 2020).

## Discussion

The coronavirus-19 disease (COVID-19) pandemic is causing economic problems for those individuals whose livelihoods have been affected due to the lockdowns occurring in many countries around the world including Bangladesh (Banna 2020). Bangladesh is beset with widespread corruption and extreme politicization alongside other issues such as money laundering which seriously hamper smooth governance and economic growth (Daily Star 2020b; Khan and Islam 2015). Consequently, the country has substantial income inequality throughout (Mazid 2019). The country is developing day by day although the wealth distribution is imbalanced. Therefore, a significant minority of individuals live below the poverty line (i.e., 20% live below the poverty line and 10.5% live in extreme poverty as reported in the 2018–2019 economic year; Financial Express 2019). Additionally, (i) the country is also ranked as having the second most unemployed graduates among Asia-Pacific countries, based on the International Labour Organization (ILO) report (Daily Jugantor 2019); (ii) youth unemployment rates doubled between 2010 and 2017 (Daily Jugantor 2019); (iii) the country has an unemployment rate of 4.4% among the general population (Daily Jugantor 2019); and (iv) 70% of the people in Bangladesh live from hand-to-mouth (Kamruzzaman 2020). However, a recent report showed extreme economic fallout due to COVID-19 crisis among poor Bangladeshi people. More specifically, per capita income dropped by 82% to $0.32 (US) in early April from $1.30 in February among individuals who live in slums compared to a 79% reduction among rural poor people (i.e., $0.39 down from $1.05; Kamruzzaman 2020).

Furthermore, sufficient food availability, production, and supply have been disrupted due to the lockdown. This has led to rising food costs making it difficult for unprivileged individuals to survive. Although the government is trying to support these people and combat the situation by the introduction of financial aid (Daily Bangladesh 2020), corruption and mismanagement have occurred during the distribution of relief goods (e.g., food, sanitary goods, household items, medicines that are required for the everyday life) and individuals have not been getting basic things they need. There have been serious allegations reported in the Bangladeshi press media (Daily Star 2020b) including the stealing and retention of relief goods by local government representatives instead of supplying it to individuals most in need. Consequently, the sudden economic recession has led needy individuals to contemplate suicide. Globally, it is well-established that unemployment, poverty, and economic distress are associated with suicide, and that when there are increases in these, there are increases in suicide (Goldman-Mellor et al. 2010; Oyesanya et al. 2015). Therefore, the suicide-related cases that are reported here are not unexpected in the COVID-19 lockdown situation because of the economic instability and disruption throughout the country.

It is worth mentioning that Bangladesh is predicted by the Asian Development Bank to face an overwhelming economic impact as a result of the COVID-19 pandemic. For instance, the country is expected to lose approximately $3 billion in GDP (i.e., 1.10% total decline) and there will be job losses for around nine million people (Banna 2020). The situation is also getting worse day by day. For instance, at the time of writing, 78,000 garment workers have protested because their wages have not been paid. (RisingBD 2020b). Additionally, the country is also losing foreign money from garment and leather product exportation, and is losing investment in large-scale projects from host countries like China (Banna 2020). Consequently, it is evident that Bangladesh is going to face great economic fallout if the lockdown persists for a long time.

There are at least eight COVID-19 suicide cases in Bangladesh (seven reported here and one previously reported by Mamun and Griffiths 2020a) and all but one was due to the economic-related issues. Additionally, the actual suicide incidence may arguably be higher than the reported cases because families do not want the death of loved ones reported as suicide news in Bangladesh to avoid the suicide-related social and criminal complexities (Mamun and Griffiths 2020d; Mamun et al. 2020a, 2020b). However, the findings for Bangladesh, a developing country, reflect the extreme psychological impacts for poor and unprivileged people.
